# Renewal of wheat cultivars enhances ozone resistance in yield but detrimentally impacts quality: a survey of Chinese wheat

**DOI:** 10.3389/fpls.2024.1526846

**Published:** 2025-01-29

**Authors:** Yinsen Qian, Zheng Zhao, Yifan Cao, Quan Ma, Nanyan Zhu, Lingqi Song, Min Zhu, Chunyan Li, Jinfeng Ding, Wenshan Guo, Xinkai Zhu

**Affiliations:** ^1^ Jiangsu Key Laboratory of Crop Genetics and Physiology, Agricultural College of Yangzhou University, Yangzhou, China; ^2^ College of Animal Science and Technology, Yangzhou University, Yangzhou, Jiangsu, China; ^3^ College of Environmental Science and Engineering, Yangzhou University, Yangzhou, Jiangsu, China; ^4^ Co-Innovation Center for Modern Production Technology of Grain Crops, Yangzhou University, Yangzhou, China; ^5^ Joint International Research Laboratory of Agriculture and Agri-Product Safety, The Ministry of Education of China, Yangzhou University, Yangzhou, China

**Keywords:** O_3_, FACE, wheat, yield, quality

## Abstract

The aggravation of ozone (O_3_) pollution poses a significant threat to agricultural production. With China being the leading wheat producer of the world, contributing 17.8% to global output, the vulnerability of wheat to O_3_ is of particular concern. Despite extensive research on the impacts of O_3_ on wheat production and the ongoing development of new wheat cultivars over the years, a connection between yield loss and the released ages of wheat cultivars under O_3_ stress remains unestablished. Addressing this, the experiment was carried out at the Yangzhou Rice and Wheat Free-air Gas Concentration Enrichment (FACE) Testing Base in China, using 17 wheat cultivars developed since the 1970s as experimental materials. The elevated O_3_ concentration in the test was 1.5 times higher than that in a normal atmosphere. The results indicated that O_3_ led to a significant reduction in wheat yield of 18.19%. The yield of cultivars released in the 1970s, 1980s, 1990s, and after 2000, decreased by 24.9%, 23.3%, 19.8%, and 14.7%, respectively. Overall, the direct effect of 1,000-grain weight on yield was the most significant, followed by the number of grains per spike, whereas the number of spikes contributed least to the yield components. To enhance resistance to O_3_ stress in future breeding efforts, increasing the 1,000-grain weight should be a primary objective. Our findings also revealed that elevated O_3_ concentration led to higher sedimentation values and protein content while lowering bulk density, hardness, and starch content. As the release age approaches, the rate of decrease in bulk density diminishes gradually. In terms of hardness, sedimentation value, and starch content, varieties released in the 1990s exhibited less sensitivity, whereas those released after the 2000s experienced the most significant changes in protein content. It is worth noting that the impact on the nutritional quality of modern cultivars is particularly significant, particularly regarding starch and protein content. Stress indices indicate that the cultivars released after 2000 exhibit stronger resistance to yield loss. The Yangmai series cultivars appear to be promising parental lines for future breeding programs aimed at developing O_3_-resistant wheat.

## 1 Introduction

Ground-level ozone (O_3_) is a widespread secondary air pollutant found in many regions worldwide and is regarded as the most significant phytotoxic pollutant in the atmosphere (Crutzen et al., 1999; Yadav et al., 2021). Its detrimental impacts on human health and ecosystems are profound and escalating (Guan et al., 2021). Although global policies have been instituted to stem the tide of rising O_3_ concentration, projections suggest that levels in the Northern Hemisphere may continue to rise by 0.5%−2% annually over the coming decades (Solomon et al., 2007). This trend signals that atmospheric O_3_ will remain a formidable challenge to the vigor of our society for the foreseeable future.

O_3_ enters plant leaves through stomata, diminishing stomatal conductance (Burkart et al., 2014), causing oxidative damage to cells, impeding various intracellular enzymatic functions (Tomer et al., 2015), and depleting chlorophyll content. These effects impair photosynthesis, culminating in substantial decrements in crop yields and quality (Burkey and Carter, 2009; Yadav A. et al., 2019). According to model estimates, global crop losses are projected to reach between $17 billion and $35 billion annually by 2030, with wheat yield losses ranging from 5.4% to 26% (Avnery et al., 2011). Mills et al. (2007) established the response function of crops to O_3_ dose in Europe and found that wheat is more sensitive to O_3_ compared with rice and maize. Even at the lower O_3_ exposure level, with an average concentration of 43 ppb, the wheat grain yield was significantly reduced by 18% (Feng et al., 2019). The damage caused by O_3_ in East Asia is even more noteworthy (Granier et al., 2011; Feng et al., 2022). In China, rapid economic and social development in recent years has resulted in environmental degradation and an accelerated rise in O_3_ concentration compared with other countries (Wang YX. et al., 2012). The concentration of surface atmospheric O_3_ in China has already reached 41 ppb and is increasing at an annual rate of 3 ppb (Wang et al., 2019). In 2017, wheat yield losses in the North China Plain were recorded at 30.8%, whereas Henan Province recorded a loss of 14.1% in 2018 (Hu et al., 2020; Wang et al., 2021). This poses a significant challenge to agriculture, making it crucial to select cultivars that can adapt to elevated O_3_ concentration in order to achieve stable yields (Yadav A. et al., 2019).

Currently, global production growth is primarily driven by genetic improvement of new cultivars and agricultural practices aimed at enhancing yield (Ding et al., 2020). Since the 1960s, genetic improvements have played a crucial role in wheat production, leading to increased grain yields in many regions (Novoselovic et al., 2000; So et al., 2022). The yield increases attributed to genetic enhancement are largely due to a rise in the number of grains per spike and an increase in individual grain weight (Tian et al., 2011). These two factors are also key components of wheat yield affected by O_3_, as highlighted in several previous studies (Chaudhary et al., 2021; Pleijel et al., 2006). The breeding goals for wheat in China have continually evolved in response to changing demands. Earlier research on wheat breeding primarily focused on disease resistance, stress tolerance, and subsequently high yield (Li et al., 2019). However, under the ongoing changes in climate conditions, enhancing stress resistance and yield through ongoing breeding efforts is essential. Some studies have found that genetic improvement promotes increased nitrogen use efficiency in many wheat cultivars (Ding et al., 2023; Fatholahi et al., 2020), a characteristic that often leads to higher yields. By verifying the evolution of agronomic traits and the physiological basis of grain yield, breeders and agronomists can develop new wheat cultivars that achieve both stability and high yield (Tian et al., 2011).

O_3_ not only affects crop yield but also impacts crop quality. Environmental conditions during the grain filling period can influence the accumulation of starch and protein, as well as their functional characteristics, including dough rheology and baking quality. In particular, environmental conditions following flowering have a significant effect on the physical properties of grains, such as their milling characteristics (Guttieri et al., 2001; Taheri et al., 2021). Numerous studies have reported that O_3_ affects not only the appearance and quality of food grains but also the mineral content, and even the health of consumers (Tomer et al., 2015, Wang YX. et al., 2012; Tripathi and Agrawal, 2012; Frei et al., 2012). Previous literature has extensively examined the impact of elevated O_3_ concentration on wheat quality (Zhang et al., 2013; Tomer et al., 2015; Yadav et al., 2020). Many researchers suggest that whereas the protein content in wheat may increase, the starch content tends to decrease. This decline is attributed to the accelerated senescence under O_3_ stress, which shortens the time available for carbohydrate synthesis in grains (Wang YX. et al., 2012). Moreover, O_3_ stress leads significant changes in the protein composition, starch granule size distribution, and a reduction in the activity of related enzymes (Wang and Frei, 2011). Many studies have still observed a decline in protein content (Mishra et al., 2013; Zhang et al., 2014); others have even found that while O_3_ may not significantly affect yield, it does lead to a deterioration in quality (Sawada et al., 2016). However, there has been limited research examining the quality of cultivars released in different decades under genetic enhancement, particularly with regard to climate change and elevated O_3_ concentration. To date, there is no unified conclusion regarding the effects of O_3_ on quality due to the complexity of quality, which involves nutrient absorption, utilization, and transformation. Further research is still needed to clarify these effects.

Prior research assessing the effect of O_3_ on wheat cultivar performance predominantly used open-top chambers (OTC) (Wahid et al., 1995; Temmerman et al., 2007; Tomer et al., 2015). However, these setups often failed to fully replicate the natural growth conditions of wheat and possessed significant limitations. Moreover, previous investigations into the effects of O_3_ exposure on wheat typically focused on locally prevalent cultivars, leaving a gap in research regarding cultivars released at different times. Our study employed the free air concentration enrichment (FACE) system, which provides a completely open to the atmospheric environment. This system ensures that other conditions such as light, temperature, water, and fertilizer same as the surrounding environment, allowing for a more accurate reflection of the impact of elevated O_3_ concentration. The utilization of an open natural field environment also eliminates the influence of numerous indoor factors and enables precise measurement of crop yield (Hu et al., 2021, 2024). We selected representative wheat cultivars popularized in the middle and lower reaches of the Yangtze River since the 1970s as materials. The primary objectives of this study were to (i) explore the relationship between the tolerance of cultivars to O_3_ stress and their releasing years; (ii) verify whether the quality parameter of O_3_-induced yield loss of wheat cultivars released in different ages was consistent; and (iii) provide suggestions for breeding work based on the mechanism of O_3_ damage.

## 2 Materials and methods

### 2.1 Experiment site and weather conditions

The experiment was conducted from 2011 to 2013 in Xiaoji Town, Jiangdu County, Jiangsu Province, China (32°35′N, 119°42′E). In this region, the traditional crop cultivation patterns are rice–wheat or rice–rape rotations. During the experiment, the area has a subtropical marine climate, characterized by a mean annual precipitation of 980 mm, a mean annual evaporation of over 1,100 mm, a mean annual temperature of 14.9°C, a total annual sunshine time of 2,100 h, and an annual frost-free lasting 220 days. The soil in the experimental field is Shajiang Aquic Cambosols, with a sandy–loamy texture. The nutrient contents in the surface layer of the soil (0 cm–20 cm) are as follows: total N 14.4 g kg^−1^, available N 70.55 mg kg^−1^, available P 11.2 mg kg^−1^, and available P 68.23 mg kg^−1^.

### 2.2 O_3_-FACE fumigation platform

The O_3_-FACE fumigation platform has been described in detail in previous studies (Zhu et al., 2011; Zhang et al., 2013). In brief, the O_3_ fumigation platform has four experimental plots (elevated [O_3_], E-O_3_) and four control plots (ambient [O_3_], A-O_3_). In the E-O_3_ plots, wheat was grown within octagons with a diameter of 14 m, surrounded by eight ABS pipes measuring 6 m each. O_3_ gas (A mixture of 5% O_3_ and 95% O_2_ produced by the KCF O_3_ generator) is injected into the center of the plot through these pipes.

**Figure 1:**
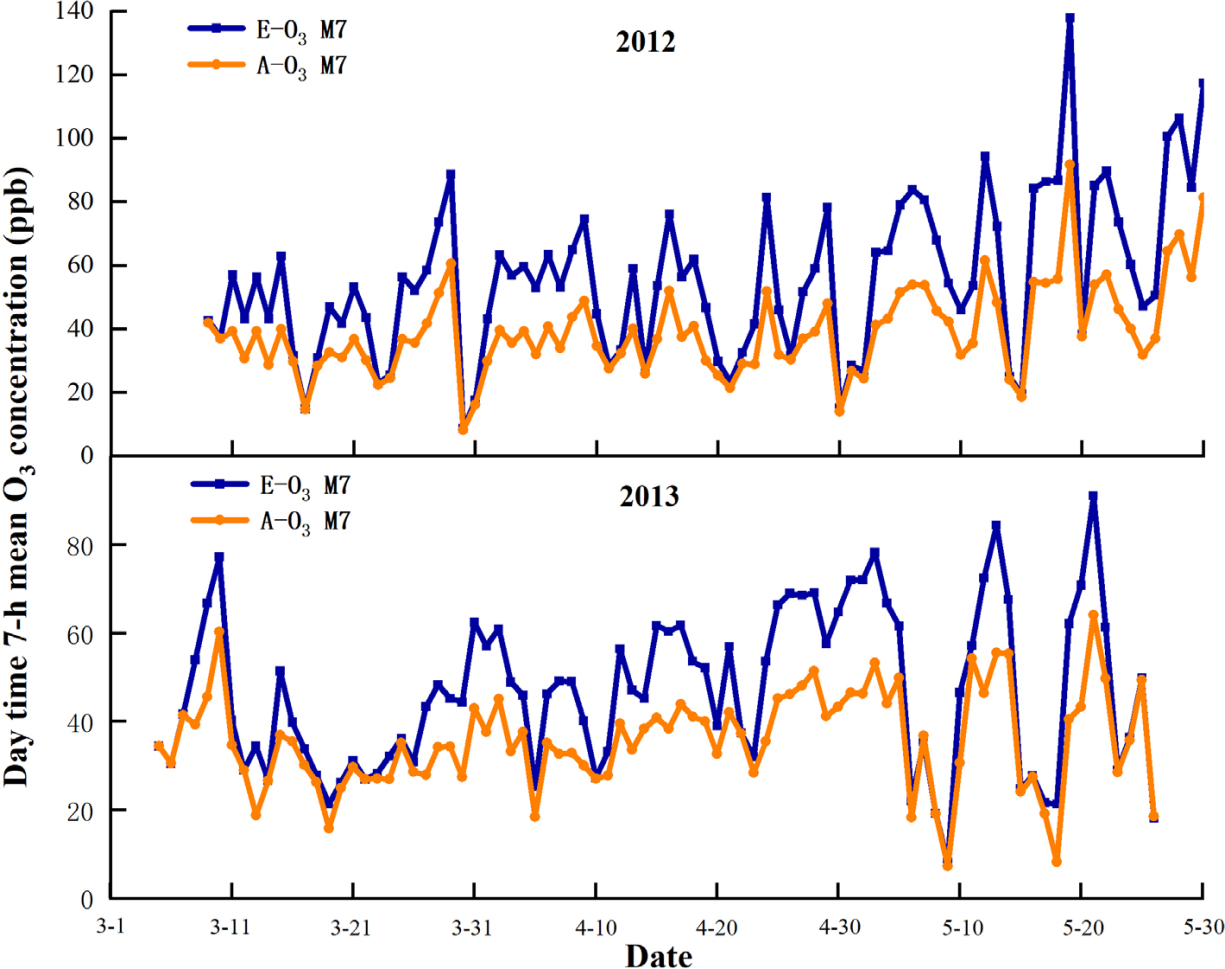
With the change in atmospheric O_3_ concentration, the average concentration of O_3_ in A-O_3_ control plots, ambient [O_3_]) and E-O_3_ circles (experimental plots, elevated [O_3_]) for 7 hours (9:00−16:00 Chinese Standard Time) per day in 2012 and 2013.

The computer controls the O_3_ concentration in the FACE circle, making the O_3_ concentration of the E-O_3_ circle 1.5 times higher than that in the A-O_3_ circle. The O_3_ fumigation lasted from 9 AM to 4 PM. Ventilation will be halted on rainy or foggy days, as well as when the ambient O_3_ concentration falls below 20 ppb or exceeds 170 ppb. A-O_3_ plots remain in the same natural state without O_3_ fumigation. The experiment experienced two growing seasons of wheat, with the O_3_ fumigation conducted from March 8 to May 29 in 2012, and from March 4 to May 25 in 2013 (**Figure 1**). In 2012, the average daily concentration of O_3_ in the E-O_3_ plots was recorded at 54.78 ppb, whereas the A-O_3_ plots had an average daily concentration of 38.93 ppb. Similarly, in 2013, the E-O_3_ plots had an average daily concentration of 46.85 ppb, whereas the A-O_3_ plots showed an average daily concentration of 36.18 ppb.

### 2.3 Plant material and cultivation

We selected a total of 17 representative wheat cultivars that have been widely cultivated in the winter wheat-growing regions of the middle and lower reaches of the Yangtze River since 1970. These cultivars include those released in the 1970s (Yangmai 1), cultivars released in the 1980s (Yangmai 3, Yangmai 4, Yangmai 5), cultivars released in the 1990s (Yangmai 6, Yangmai 158, Yangmai 9, Yangmai 10), and cultivars released after 2000 (Yangmai 11, Yangmai 12, Yangfumai 2, Yangmai 13, Yangmai 14, Yangmai 16, Yangmai 15, Yangmai 19, Yangmai 20) (**Table 1**).

**Table 1:** Approval time, pedigree, and character traits of each cultivar of the Yangmai winter wheat series.

Cultivar	Approval time and province	Pedigree	Character traits
Yangmai 1	1967, Jiangsu	Funo Series	*
Yangmai 3	1983, Anhui	Yangmai 1 Series	Late sowing resistant
Yangmai 4	1982, Jiangsu	Nanda 2419/Shenglimai×Axuan 2	Early maturity
Yangmai 5	1986, Jiangsu	Nanda 2419/Shenglimai//Funo×St1472/506	High plant height
Yangmai 6	1991, Jiangsu	Dafeng 1087×Zaoshu 5	Sturdy stalk and strong lodging resistance
Yangmai 158	1993, Jiangsu	Yangmai 4×St1472/506	High spikelet setting rate
Yangmai 9	1996, Jiangsu	Jiansan ×Yangmai5	Strong lodging resistance and cold resistance
Yangmai 10	1998, Jiangsu	Yang5×Y.C/Jiansan/3/Yangmai 158	Excellent quality and strong disease resistance
Yangmai 11	2000, Jiangsu	Yangmai 158/3/Y.C/Jiansan×Yang 85−85	Strong lodging resistance
Yangmai 12	2001, Jiangsu	Yangmai 158/3/TP114/Yangmai 5×Yang 85−85	Damp resistant and strong powdery mildew resistance
Yangfumai 2	2002, Jiangsu	Yangmai 158×101−901	Frost resistant, strong lodging resistance and leaf rust resistance
Yangmai 13	2003, Jiangsu	Yang 88−84×Maris Dove/Yangmai 3	Fast grain filling rate, sturdy stalks and high spike formation rate
Yangmai 14	2004, Jiangsu	Yangmai 158×Yangmai 6	Fertility tolerant and lodging resistant
Yangmai 15	2005, Jiangsu	Yang 89−40×Chuanyu 21526	Fertility tolerant and lodging resistant,
Yangmai 16	2004, Jiangsu	Yang 91F138×Yang 90−30	Frost resistant and strong tillering capacity
Yangmai 19	2008, Anhui	6×Yangmai 9/4/4×158/3/4×Yang 85−85//Yangmai 5/(Yuma/8×Chancellor)	powdery mildew resistance and strong tillering capacity
Yangmai 20	2012, Jiangsu	Yangmai 10×Yangmai 9	strong tillering capacity

*The related cultivar characteristics of Yangmai1 cannot be queried, because the cultivar is too old.

The seeds were manually sown at a planting density of 2.25 million ha^−1^ (24,300 plants per circle), with a row spacing of 25 cm. The fertilizer application amount and schedule were consistent across all experimental plots. Nitrogen fertilizer was applied as urea (N=46%) and at a total rate of 210 kg N ha^−1^, which was applied in three stages: pre-sowing, five-leaf stage, and jointing stage with a ratio of 6:1:3. The phosphorus and potassium fertilizers were applied at rates of 90 kg P_2_O_5_ ha^−1^ and 90 kg K_2_O ha^−1^, respectively. Of these, 60% of the phosphorus and potassium fertilizers were applied at the planting stage, whereas the remaining 40% was applied during the jointing stage.

### 2.4 Sampling and chemical analyses

We evaluated the yield and its components of wheat planted in 2012 and 2013 and assessed some quality parameters after the harvest in 2013. The specific indicators and measurement methods are as follows.

#### 2.4.1 Grain yield and its components

For the determination of actual grain yield, a total of 2 m² of plants located away from the border of each plot was harvested at maturity. To determine the number of grains per spike, 50 consecutive spikes were examined in the field. 1 m^2^ in the center of each plot was randomly selected to calculate the number of spikes, which were then separated and dried after harvest. To determine the 1,000-grain weight, 1,000 grains were selected and weighed. The above figures on grain and its composition have been repeated four times.

#### 2.4.2 Milling quality parameters

We selected the spike harvested at maturity and removed the stems and glumes to obtain the grains. The grain bulk density is measured using the HGT-1000 bulk density instrument (Dongfang Scales Corporation, Shanghai, China). At the same time, the hardness of the grains is determined using the hardness tester (Sanfeng Corporation, Guangzhou, China).

#### 2.4.3 Sedimentation value

Take grains harvested at maturity and grind them into flour using a Brabender mill D-28033 (Brabender Corporation, Duisburg, Germany). Measure out 3.2 g of the flour and place it into a 100-mL graduated cylinder. Add 50 mL of bromothymol blue solution (4 mg L^−1^), and shake the cylinder 12 times. Then, place it on a shaker for 5 min. Next, add 25 mL of a lactate–isopropanol mixture (prepare this by diluting 250 mL of 85% lactic acid with water to a final volume of 1 L, then measure out 250 mL and combine it with 200 mL of isopropanol, and adjust the total volume to 1 L and allow the mixture to sit for 48 h). Shake the cylinder for an additional 5 min, then let it sit for 5 min before taking the reading. The final reading, recorded to the nearest 0.1 mL, represents the sedimentation value of the flour.

#### 2.4.4 Grain starch content

Starch content is measured using the anthrone colorimetric method (Yan et al., 2022). First, accurately weigh 0.2 g of the ground sample from Section 2.1, and place it in a 15-mL test tube. Add 6 mL of 80% ethanol, and heat the sample in a water bath at 80°C for 30 min. Afterward, centrifuge at 3,000 g for 4 min and discard the supernatant. Repeat this process three times, then dry the precipitate in an oven. Transfer it to a 50-mL culture tube containing 20 mL of distilled water and place the mixture in a boiling water bath for 15 min. After cooling, add 2 mL of 9.2 mol L^−1^ perchloric acid while stirring continuously, and dilute with distilled water to a final volume of 10 mL. Centrifuge for 10 min, and pour the supernatant into a 50-mL volumetric flask. Repeat this step twice and then make up to the mark. Adjust the optical density (OD) measurement at a wavelength of 625 nm using a blank for calibration, and determine the starch content based on a standard curve.

#### 2.4.5 Grain protein content

Pass the samples from Section 3.1 through a 2-mm sieve. The nitrogen content of the grains is measured using the H_2_SO_4_–H_2_O_2_ digestion method and the micro-Kjeldahl procedure method (Douglas et al., 1980). The protein content is calculated by multiplying the nitrogen content by 5.7.

#### 2.4.6 Yield stress indexes

The estimation of the Stress Sensitivity Index (SSI) is based on the calculations by Fischer and Maurer (1978).

The calculation method for the Stress Tolerance Index (STI) refers to Kristin et al. (1997).

The Geometric Mean Productivity (GMP) was calculated according to Fernandez (1992).

### 2.5 Statistical analysis

Excel 2016 (Microsoft Corporation, Washington, USA) and SPSS 24.0 (Microsoft Corporation, Washington, USA) were used for data sorting and analysis of this experiment, and Origin 2018 (OriginLab, Northampton, USA) was used for creating charts. Analysis of variance (ANOVA) was employed to assess the level of difference between the cultivars. A two-way ANOVA was conducted to examine the main and interaction effects of O_3_ and cultivar on yield, yield components, and quality parameters. The reduction rates of wheat cultivars from different ages under elevated O_3_ concentrations were analyzed using one-way ANOVA. Duncan multiple range test was performed for mean comparisons, with *p* < 0.05 considered statistically significant. Based on the consistent trends in yield and its components, the Results section uses 2-year average values for descriptive analysis. A linear regression model was employed for path analysis, using the yield of E-O_3_ treatment as the dependent variable, spike number, grains per spike, and 1,000-grain weight of E-O_3_ treatment as independent variables.

## 3 Results

### 3.1 Effect of elevated O_3_ concentration on grain yield

**Figure 2:**
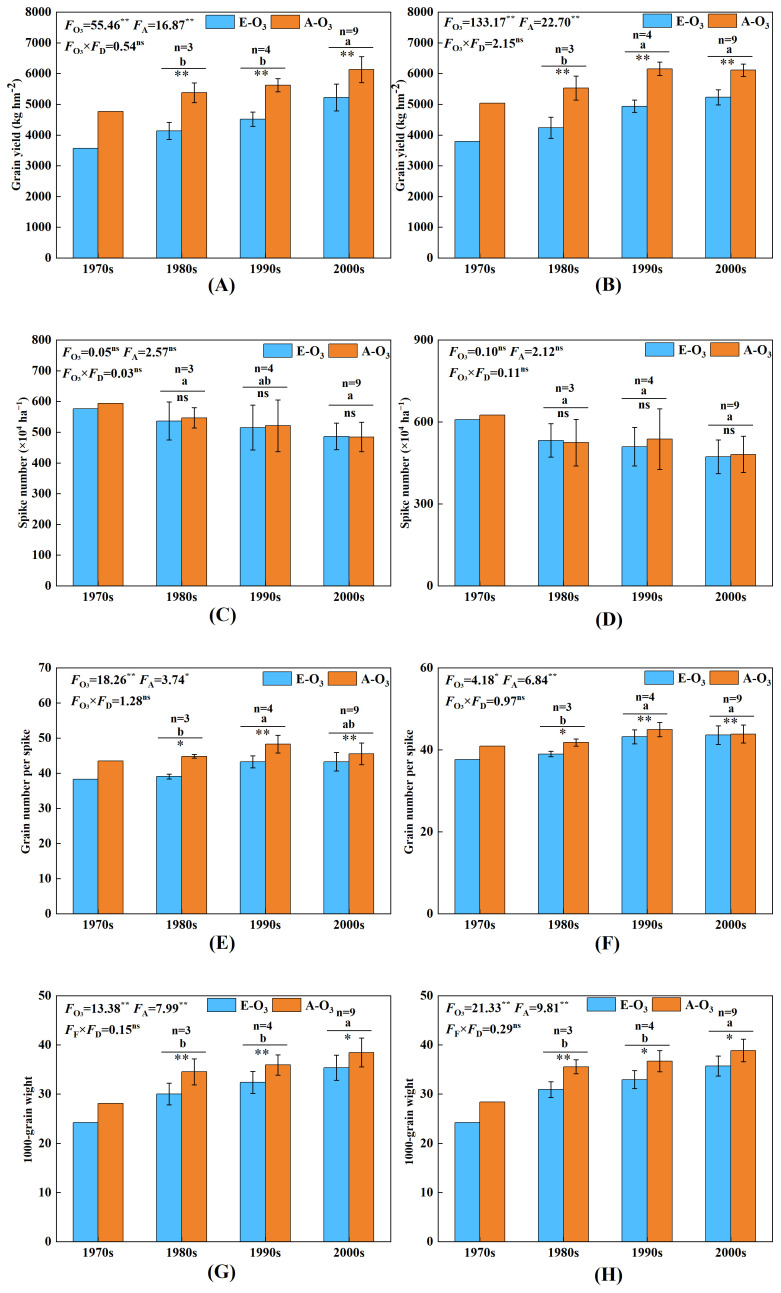
Effect of elevated O_3_ concentration on yield and its composition of wheat cultivars released in four ages. E-O_3_, elevated O_3_ concentration treatment; A-O_3_, normal atmospheric control. Different lowercase letters represent significant differences between cultivars (*p* < 0.05). ** indicates a mean significance level of *p* < 0.01, * indicates a mean significance level of *p* < 0.05, whereas ns indicates a mean significance level of *p* > 0.05 and is not significant. The markings on the error bars indicate the significance of the effect of O_3_ on the same age cultivars. The results of the cultivar released in the 1970s are for reference only and are not included in the variance analysis. **(A, B)** represent the experimental results of yield for the years 2012 and 2013, respectively. **(C, D)** represent the experimental results of spike number, and **(E, F)** represent the experimental results of grain number per spike. **(G, H)** represent the experimental results of the 1,000-grain weight. **(A, C, D, G)**, represent the experimental results from 2012; **(B, E, F, H)** represent the experimental results from 2013.

**Figure 3:**
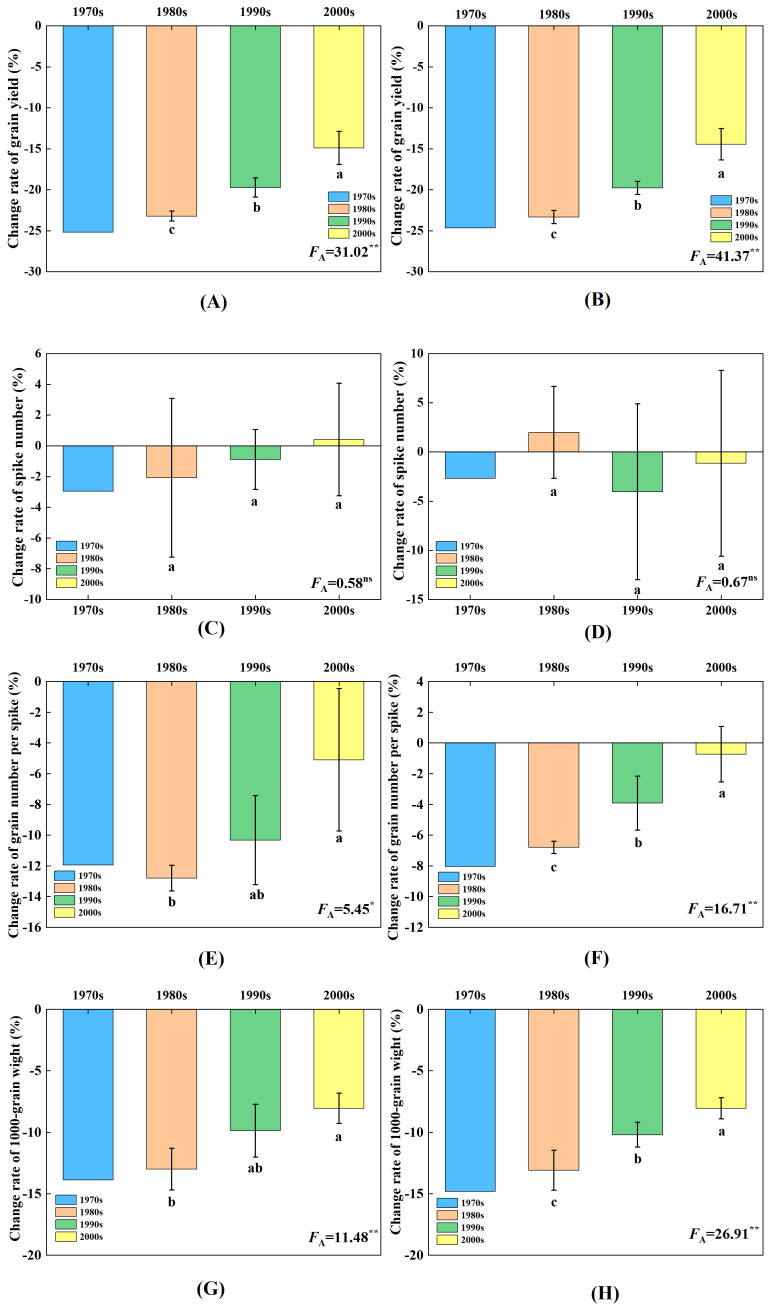
Effect of elevated O_3_ concentration on yield and its composition loss rate of wheat cultivars released in four ages. Different lowercase letters represent significant differences between cultivars (P < 0.05). * and ** indicate a mean significance level of <0.05 and significance level p <0.01, respectively; ns means not significant. **(A, B)** represent the experimental results of yield loss rate for the years 2012 and 2013, respectively. **(C, D)** represent the experimental results of spike number loss rate, and **(E, F)** represent the experimental results of grain number per spike loss rate. **(G, H)** represent the experimental results of the 1,000-grain weight loss rate. **(A, C, D, G)**, represent the experimental results from 2012; **(B, E, F, H)** represent the experimental results from 2013.

Compared with the A-O_3_ treatment, the E-O_3_ treatment significantly decreased the wheat grain yield in both years (**Figures 2A, B**). The yield reduction ranged from 759.4 kg ha^−1^ to 1,338.5 kg ha^−1^, with an average reduction of 1,043.7 kg ha^−1^. The yield decrease ranged from 12.3% to 25.2%, with an average decline of 17.9%, also displaying a significant difference (**Figures 3A, B**).

Under the condition of elevated O_3_ concentration, there were differences in yield reduction among wheat cultivars released in different years, and these variations reached significant levels. The yield of wheat cultivars released in the 1970s, 1980s, 1990s, and after 2000 decreased by 24.9%, 23.3%, 19.8%, and 14.7%, respectively (**Figures 3A, B**). These results showed that the older the wheat cultivars released with the elevated O_3_ concentration, the larger the grain yield decreased.

### 3.2 Effect of elevated O_3_ concentrations on spike number

Compared with the control A-O_3_ treatment, the E-O_3_ treatment showed both increased and decreased changes in spike number in certain cases (**Figures 2C, D**). The changes ranged from −73.6×10^4^ to 67.2×10^4^ ha^−1^, with an average decrease of 6.67×10^4^ ha^−1^, but these differences were not significant. The range of change in spike number was between −10.7% and 14.8%, with an average decrease of 1.0%, and the difference was not significant (**Figures 3C, D**).

The elevated O_3_ concentration had no significant effect on the panicle number of wheat cultivars released in different years. The spike number of wheat cultivars released in the 1970s, 1980s, 1990s, and after the 2000s showed changes of −2.8%, −0.1%, −2.5%, and −0.4%, respectively (**Figures 3C, D**).

### 3.3 Effect of elevated O_3_ concentration on grain number per spike

Compared with the control A-O_3_, the E-O_3_ treatment in both years significantly affected the number of grains per spike (**Figures 2E, F**), resulting in a decrease of 7.4 to −1.2 grains per spike, with an average reduction of 2.5 grains per spike. From (**Figures 3E, F**), we observed that the number of grains per spike decreased from 14.5% to −2.6%, with an average reduction of 5.3%.

The effect of elevated O_3_ concentration on the variation of grain number per spike of wheat cultivars released at different ages was different, the cultivars released in the 1990s and 2000s were significantly higher than those in the 1980s. Under the condition of elevated O_3_ concentration, the cultivars released in the 1970s, 1980s, 1990s, and after the 2000s, the number of grains per spike decreased by −10.0%, −9.8%, −7.1%, and −2.9%, respectively (**Figures 3E, F**).

### 3.4 Effect of elevated O_3_ concentration on the 1,000-grain weight

In our 2-year experiments, the 1,000-grain weight showed a significant decrease of 2.1 g−5.4 g, with an average decrease of 3.6 g (**Figures 2G, H**). The decrease was 6.0%−14.8%, with an average reduction of 9.8%, and the difference reached a significant level (**Figures 3G, H**).

The effect of elevated O_3_ concentration on the variation in 1,000-grain weight differed among wheat cultivars released at different times, and this effect was found to be significant. For the cultivars released in the 1970s, 1980s, 1990s, and after 2000, the 1,000-grain weight decreased by −14.4%, −13.1%, −10.1%, and −8.1%, respectively (**Figures 3G, H**).

### 3.5 Path coefficient analysis

The path coefficient analysis revealed that the direct path coefficient order of yield components on grain yield in the 2-year experiment was 1,000-grain weight > grains per spike > spikes, suggesting that the contribution of yield components to grain yield followed the order of 1,000-grain weight > grains per spike > spikes (**Table 2**).

**Table 2:** Path coefficient analysis showing direct and indirect effects of yield components on grain yield of wheat released in different years.

Year	Age	Yield component	Correlation coefficient with yield	Direct path coefficient	Indirect path coefficient		
					Spikes	Grains per spike	1000-grain weight
2012	ALL	Spikes	0.009	0.065	−	−0.012	0.086
		Grains per spike	0.816	0.508	−	−	0.406
		1000-grain weight	0.852	0.562	−	−	−
	The 1970s	Spikes	0.118	0.053	−	−0.169	−0.180
		Grains per spike	0.778	0.617	−	−	0.245
		1000-grain weight	0.695	0.562	−	−	−
	The 1980s	Spikes	−0.260	0.396	−	−0.189	−0.178
		Grains per spike	0.627	0.448	−	−	0.369
		1000-grain weight	0.598	0.434	−	−	−
	The 1990s	Spikes	−0.708	0.478	−	−0.128	−0.279
		Grains per spike	0.606	0.328	−	−	0.241
		1000-grain weight	0.712	0.466	−	−	−
	After 2000	Spikes	−0.239	0.156	−	−0.063	−0.020
		Grains per spike	0.372	0.187	−	−	0.213
		1000-grain weight	0.647	0.416	−	−	−
2013	ALL	Spikes	0.051	0.111	−	−0.073	0.065
		Grains per spike	0.907	0.407	−	−	0.411
		1000-grain weight	0.924	0.546	−	−	−
	The 1970s	Spikes	−0.408	0.275	−	−0.186	−0.061
		Grains per spike	0.869	0.625	−	−	0.124
		1000-grain weight	0.482	0.386	−	−	−
	The 1980s	Spikes	0.730	0.217	−	−0.032	0.513
		Grains per spike	0.643	0.578	−	−	0.654
		1000-grain weight	0.985	0.855	−	−	−
	The 1990s	Spikes	−0.618	0.657	−	0.032	0.008
		Grains per spike	0.488	0.115	−	−	0.555
		1000-grain weight	0.777	0.692	−	−	−
	After 2000	Spikes	0.134	0.274	−	0.094	−0.065
		Grains per spike	0.231	0.139	−	−	0.216
		1000-grain weight	0.686	0.606	−	−	−

According to the path coefficient analysis, the elevated O_3_ concentration significantly reduced the number of grains per spike and 1,000-grain weight of cultivars released in different years, and the decrease was in the order of cultivars released after the 2000s < cultivars released in the 1990s < cultivars released in the 1980s < cultivars released in the 1970s. The effect of yield components on yield differed among cultivars released at different times. For cultivars released in the 1970s, the direct effect of the decrease in grain number per spike on yield was the highest, followed by 1,000-grain weight in both years.

### 3.6 Effect of elevated O_3_ concentration on the bulk density

**Figure 4:**
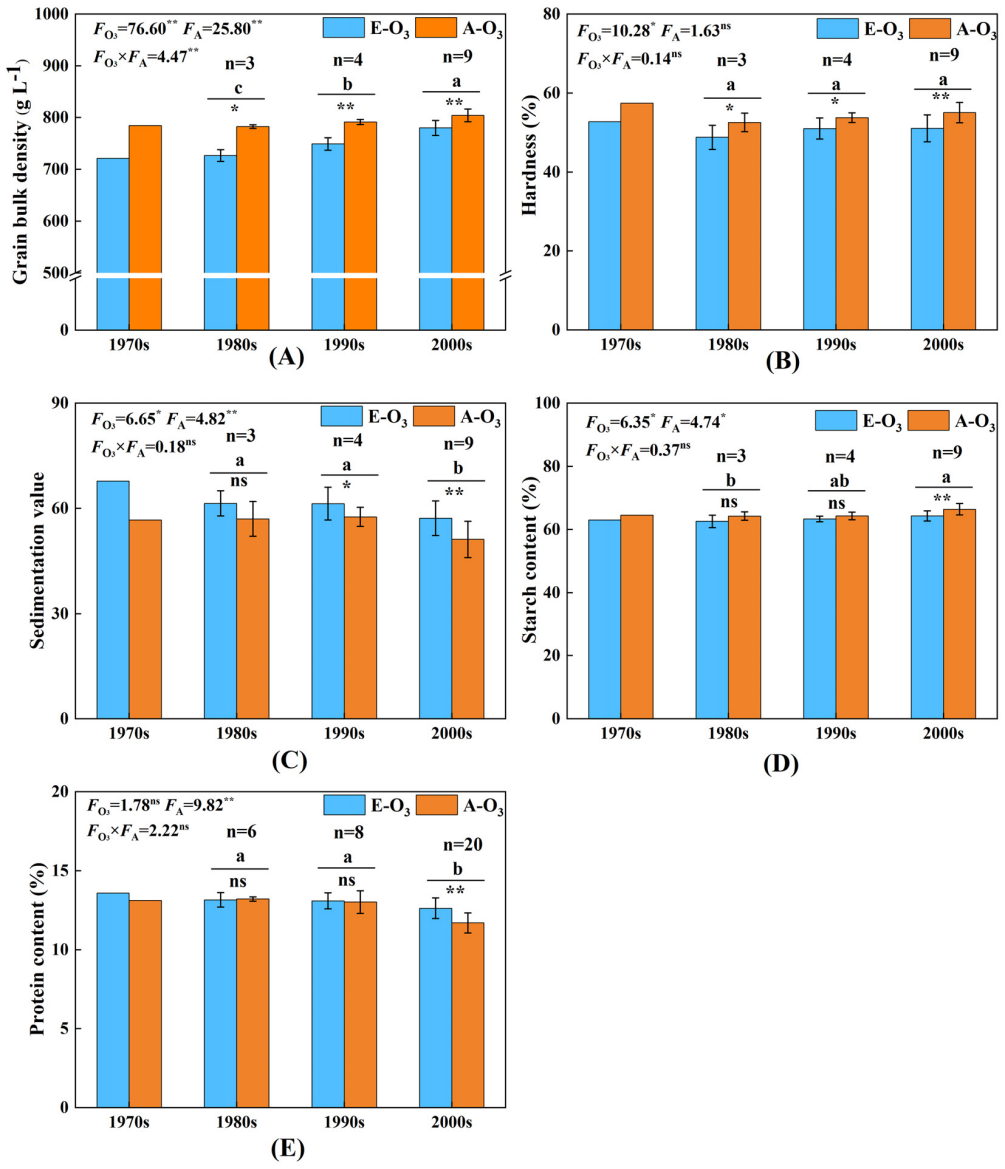
Effect of elevated O_3_ concentration on quality of wheat cultivars released in four ages. E-O_3_, elevated O_3_ concentration treatment; A-O_3_, normal atmospheric control. Different lowercase letters represent significant differences between cultivars (*p* < 0.05). ** indicates a mean significance level of *p* < 0.01, * indicates a mean significance level of *p* < 0.05, whereas ns indicates a mean significance level of *p* > 0.05 and is not significant. The markings on the error bars indicate the significance of the effect of O_3_ on the same age cultivars. The results of the cultivar released in the 1970s are for reference only and are not included in the variance analysis. **(A–E)** represent the bulk density, hardness, sedimentation value, starch content, and protein content, respectively.

**Figure 5:**
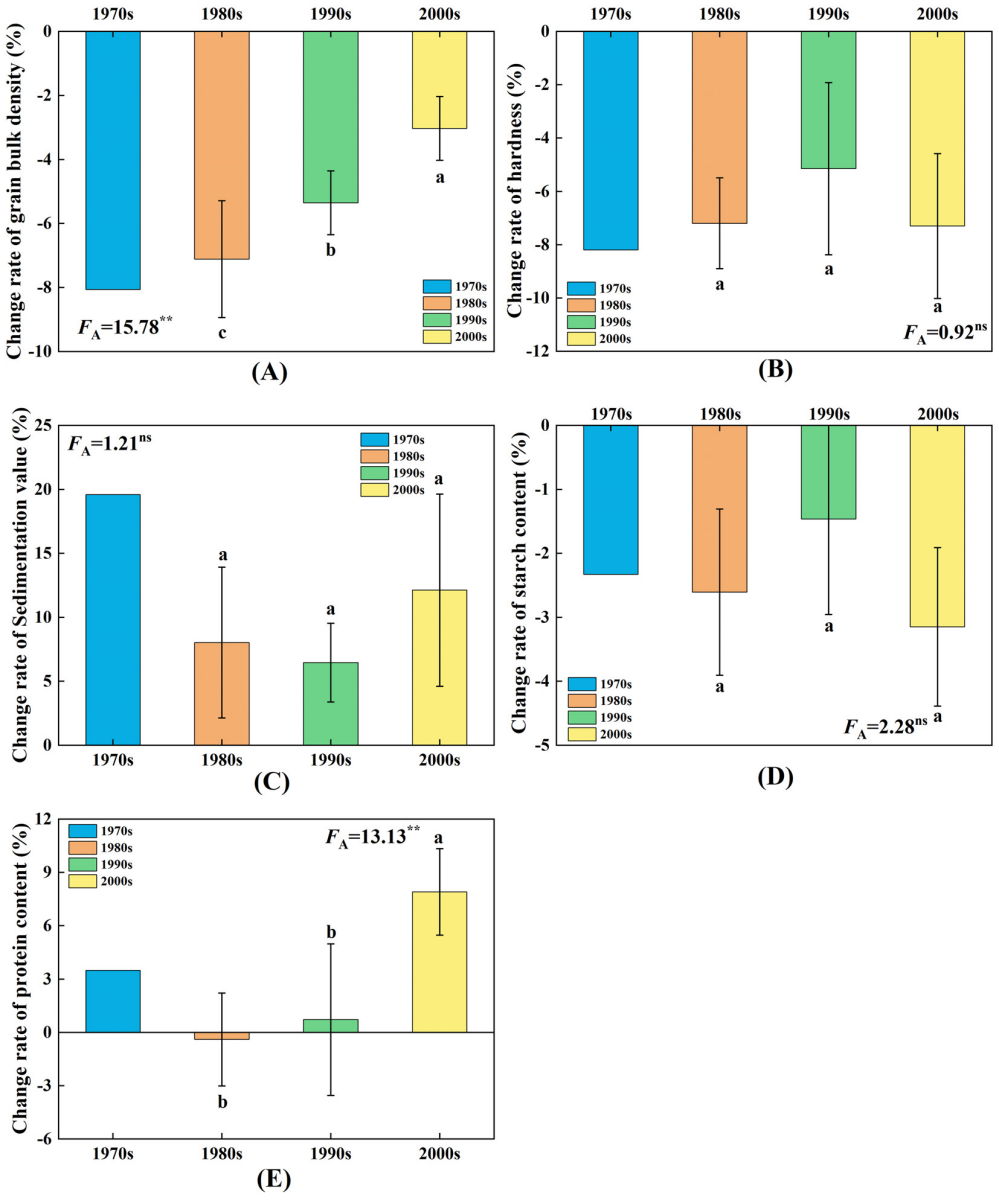
Effect of elevated O_3_ concentration on quality loss rate of wheat cultivars released in four ages. Different lowercase letters represent significant differences between cultivars (P < 0.05). **(A–E)** represent the loss rate of bulk density, hardness, sedimentation value, starch content, and protein content, respectively. ** indicates a mean significance level of *p* < 0.01, while ns indicates a mean significance level of *p* > 0.05 and is not significant.

The impact of elevated O_3_ concentration on the bulk density of wheat cultivars released in different ages is illustrated in **Figure 4A**. Elevated O_3_ concentration significantly reduced the bulk density of wheat cultivars from each age. The results of the variance analysis indicate that both the elevated O_3_ concentration and the released year of cultivars significantly affected the bulk density of wheat. Wheat cultivars released after the year 2000 had a notably higher bulk density compared with those released in the 1980s and 1990s. As shown in **Figure 5A**, the influence of O_3_ on the bulk density of wheat cultivars diminished as the release year approached. The decline rates for the different ages were 8.1%, 7.1%, 5.4%, and 3.0%, respectively, indicating that the bulk density of cultivars released in the 2000s was significantly less affected by O_3_ compared with those released in the previous two decades.

### 3.7 Effect of elevated O_3_ concentration on the hardness

The impact of elevated O_3_ concentration on the hardness of wheat cultivars released in different ages is illustrated in **Figure 4B**. Elevated O_3_ concentration significantly reduced the hardness of wheat cultivars from each age. The results of the variance analysis indicate that although elevated O_3_ concentration significantly affected the hardness of wheat, there were no significant differences among the cultivars released in different ages. As shown in **Figure 5B**, the reduction in hardness of wheat grains under elevated O_3_ treatment for different ages was 8.2%, 7.2%, 5.2%, and 7.3%, respectively, with no significant differences in the decline rates among cultivars released in different ages.

### 3.8 Effect of elevated O_3_ concentration on the sedimentation value

The impact of elevated O_3_ concentration on the sedimentation value of wheat cultivars released in different ages is illustrated in **Figure 4C**. Elevated O_3_ concentration significantly lowered the sedimentation values of wheat cultivars released in the 1990s and 2000s. The results of the variance analysis indicate that both the elevated O_3_ concentration and the released year of cultivars significantly affected the sedimentation values, with cultivars released after 2000 showing significantly lower values than those released in the 1980s and 1990s. As shown in **Figure 5C**, the reduction in sedimentation values of wheat grains under elevated O_3_ treatment for different ages was 19.6%, 8.0%, 6.5%, and 12.1%, respectively, with no significant differences in the decline rates among cultivars released in different ages.

### 3.9 Effect of elevated O_3_ concentration on the starch content

The impact of elevated O_3_ concentration on the starch content of wheat cultivars released in different ages is illustrated in **Figure 4D**. Elevated O_3_ concentration significantly reduced the starch content of wheat cultivars released in the 2000s. The results of the variance analysis indicate that both the elevated O_3_ concentration and the released year of cultivars significantly affected the starch content. Cultivars released after 2000 had significantly higher starch content compared with those released in the 1980s. As shown in **Figure 5D**, the reductions in starch content of wheat grains under elevated O_3_ treatment for different ages were 2.3%, 2.6%, 1.5%, and 3.2%, respectively, with no significant differences in the decline rates among cultivars released in different decades.

### 3.10 Effect of elevated O_3_ concentration on the protein content

The impact of elevated O_3_ concentration on the protein content of wheat cultivars released in different ages is illustrated in **Figure 4E**. Elevated O_3_ concentration significantly reduced the protein content of wheat cultivars released after 2000. The results of the variance analysis indicate that the released year of cultivars significantly affected the protein content of wheat grains. Cultivars released after 2000 had significantly lower protein content compared with those released in the 1980s and 1990s. As shown in **Figure 5E**, the influence of O_3_ on the protein content of wheat grains decreased as the release year approached, with reduction rates of 3.5%, −0.4%, 0.7%, and 7.9%, respectively. The change in protein content of cultivars released in the 2000s was significantly greater due to O_3_ compared with those released in the previous two decades.

### 3.11 Stress evaluation indices

The results from the 2 years demonstrate that the year of cultivar release significantly affects the three stress evaluation indices (**Table 3**). The values of the three indices for cultivars released in the 2000s show significant differences compared with those released in the 1980s. As the year of cultivar release approaches, the SSI gradually decreases, whereas both STI and GMP increase.

**Table 3:** Related stress tolerance index of cultivars released in different ages.

	SSI		STI		GMP	
Age	2012	2013	2012	2013	2012	2013
1970s	1.41	1.39	0.25	0.25	4.12	4.37
1980s	1.30a	1.32c	0.29b	0.28b	4.714b	4.84b
1990s	1.11b	1.13b	0.30b	0.31a	5.036b	5.51a
2000s	0.84c	0.82c	0.34a	0.32a	5.656a	5.65a
*F* test	31.07**	41.33**	8.54**	11.74**	8.85*	12.32**

SSI, Stress Sensitivity Index; STI, Stress Tolerance Index; GMP, geometric mean productivity. The results of the cultivar released in the 1970s are for reference only and are not included in the variance analysis. Different lowercase letters represent significant differences between cultivars (*p* < 0.05). * and ** indicate a mean significance level of <0.05 and signiﬁcance level p <0.01, respectively.

## 4 Discussion

### 4.1 Effects of O_3_ on the yield of wheat released at different ages

Recently, both rice and wheat have primarily been cultivated with hybrid cultivars (Ding et al., 2020; Zhu et al., 2020). It is well known that hybrids have higher productivity to meet human needs. However, under the changing global climate, recent studies have suggested that hybrids are more susceptible to O_3_ damage. Pleijel et al. (2006) examined the effects of O_3_ on a 100-year-old wheat cultivar compared with a modern wheat cultivar, finding that the old cultivar was less affected by O_3_ in terms of yield and 1,000-grain weight compared with the new cultivar. Barnes et al. (1990) evaluated 10 wheat cultivars introduced in Greece and discovered that modern cultivars had a lower relative growth rate compared with older cultivars when exposed to O_3_. Singh et al. (2018) proposed that high-yielding modern cultivars are more susceptible to O_3_ damage than older cultivars due to their high stomatal conductance, which results in increased O_3_ flux. These studies collectively suggest that despite their high yields, modern cultivars are highly sensitive to O_3_ and may face more vulnerability to higher O_3_ damage in the future. However, contrary to our expectations, our study on Yangmai series wheat cultivars subjected to O_3_ stress revealed a different pattern of yield reduction. Over the 2-year experiment, we observed that elevated O_3_ concentration significantly reduced the yield of wheat cultivars released in the 1970s, 1980s, 1990s, and after the 2000s, with average yield reductions of 24.9%, 23.3%, 19.8%, and 14.7%, respectively. These results indicate that wheat cultivars released at different times exhibited varying sensitivities to elevated O_3_ concentrations, with yield reduction rates decreasing as the release date of the cultivars progressed. This suggests that the Yangmai series wheat has demonstrated adaptability to the elevated O_3_ concentration through continuous breeding, leading to an enhanced ability to resist O_3_. We speculate that this adaptability could be linked to the atmospheric O_3_ concentration at the time of cultivar release. The middle and lower reaches of the Yangtze River are one of the main wheat-producing areas in China and also an important economic zone where O_3_ concentration has been rising (Wang XK. et al., 2012). Fusarium head blight has been the predominant disease that limited wheat production in China over the past century (Zhou et al., 2007a). The breeding of the Yangmai series began with the introduction and reselection of the Italian cultivar cv. Funo. Subsequently, the Yangmai 158 cultivar emerged as an iconic cultivar, demonstrating excellent yield stability in its release year. Many subsequent breeding programs have used it as a parent cultivar (**Table 1**), as it significantly enhanced resistance to Fusarium head blight and powdery mildew, in addition to improving tolerance to high temperatures during the grain-filling period (Zhou et al., 2007b). The tolerance of Yangmai series wheat may be enhanced with the elevated O_3_ concentration during cultivation, which suggested that the Yangmai series wheat could be a good parent for improving O_3_ resilience in wheat. Although O_3_ resistance is not explicitly considered a breeding criterion, resistance to air pollutants appears to be heritable within the same crop (Burkey et al., 2000; Fiscus et al., 2005). Consequently, we have reason to believe that breeding for high disease resistance in the Yangmai series may inadvertently enhance resistance to O_3_ as well.

In a physiological study conducted by Biswas et al. (2008) on 20 wheat cultivars of different ages, it was found that higher stomatal conductance and a greater reduced antioxidant capacity in the new cultivars led to oxidative damage to the cell membrane, resulting in increased sensitivity to O_3_. This finding may seem inconsistent with the conclusion of our experiment. We have analyzed the possible reasons for this discrepancy, focusing on the relationship between antioxidant enzyme activity and wheat resistance to O_3_. The content of antioxidant enzymes in the leaves of O_3_-sensitive and O_3_-tolerant plants may not differ significantly under O_3_ stress (Burkey et al., 2000). Additionally, the activity of antioxidant enzymes in plants varied at different growth stages under O_3_ stress, meaning that relying solely on antioxidant enzyme activity is insufficient to conclusively determine the plants’ growth status. Typically, the photosynthesis of wheat cultivars under O_3_ stress will decline; however, some new cultivars experience a lower rate of decline compared with older cultivars, which may be attributed to the repair of the plant antioxidant system (Chaudhary et al., 2021; Biswas et al., 2008). Our results demonstrate the effects of O_3_ stress on wheat in terms of yield loss; yield is a critical indicator for crops and a primary consideration for agricultural selection. Our experimental results showed that the new cultivar exhibited less yield loss than the old cultivar under O_3_ stress. After comparing the loss of photosynthetic capacity and enzyme activity of the cultivars in the relevant experiments of other researchers, the old cultivar was thought to be more capable than the new cultivar, because the higher photosynthetic rate of the new cultivars also led to higher stomatal conductance and thus the risk of absorbing more O_3_ (Biswas et al., 2008; Harmens et al., 2018). We believe that this may be related to the stress response of plants to O_3_. In the case of acute exposure, wheat plants received stress that causes a decline in photosynthesis and other physiological reactions. However, prolonged exposure to O_3_, plants can develop adaptive responses, and cultivars can adapt to the atmospheric O_3_ concentration present during their breeding period, thus enhancing relative resistance (Ojanpera et al., 1998).

In recent years, plant breeders have shown a preference for developing cultivars with improved nitrogen use efficiency and enhanced drought resistance. Earlier research has indicated a possible linkage between elevated nitrogen use and increased resistance to O_3_ damage (Velissariou et al., 1992). While some older wheat cultivars may have a lower yield than modern cultivars, they often exhibit higher resistance. This is likely because older cultivars were selected through natural planting, allowing them to strengthen their disease and stress resistance as they adapted to environmental changes (Barnes et al., 1999; Burkey et al., 2000). In contrast, modern cultivars are artificially selected with the specific goal of meeting human requirements. However, this breeding selection for desired traits may unintentionally lead to changes in tolerance to O_3_ damage (Biswas et al., 2009). Regarding the relevant stress indices SSI, STI, and GMP, a lower SSI corresponds to higher STI and GMP, indicating greater yield tolerance and stronger resistance under stress (Ghanem and Al-Farouk, 2024). In our research, we found that cultivars released after 2000 performed exceptionally well across all three indicators. This suggested that the cultivars of the Yangmai series that have been continuously artificially released can adapt to the environment with rising O_3_ concentration. Modern cultivars are more suitable for the current environment (higher CO_2_ and O_3_ levels) and management practices than older cultivars, potentially resulting in higher yield performance and improved tolerance.

### 4.2 The effect of O_3_ on the constituent factors of yield

Wheat yield is jointly determined by spike number, grain number per spike, and 1,000-grain weight. Studies have reported the effect of elevated O_3_ concentration on the component factors of yield, but the results are inconsistent. Some studies indicated that high O_3_ levels greatly reduced the seed-setting rate of wheat, leading to a decrease in the number of grains per spike (Chaudhary et al., 2021; Tomer et al., 2015), whereas other studies have highlighted that elevated O_3_ concentrations notably impact the seed-setting rate of winter wheat. For instance, Pleijel et al. (2006) believed that elevated O_3_ concentration slightly reduces the number of grains per spike; although not to a significant extent, it does reduce the 1,000-grain weight of wheat. However, the decrease in grain weight under our results indicates that the 1,000-grain weight is particularly affected when the O_3_ concentration increases in the FACE system. These findings are consistent with the study conducted by Fangmeier et al. (1994) and Zhang et al. (2014) under FACE conditions. Path analysis revealed that the decrease in 1,000-grain weight had the most significant impact on reducing wheat yield, whereas the number of spikes had a minimal effect. In contrast, some studies on Indian wheat have found that O_3_ frequently impacts the number of spikes, resulting in a decrease in yield (Mishra et al., 2013; Yadav et al., 2020). The impact may be attributed to varying O_3_ concentrations during the different growth stages of wheat. In China, before the jointing stage (a critical period for spike development), when the atmospheric temperature is low, the O_3_ concentration is minimal and has little influence on spike numbers. However, after the jointing stage (a critical period for grain formation and number), O_3_ concentration levels increases with rising temperature, resulting in a greater impact on wheat. During the spike differentiation process of wheat in India, high temperatures and elevated O_3_ concentrations directly caused damage to the number of spikes (Xu et al., 2024). The varying results in yield composition among different cultivars from different ages may be attributed to differences in varietal characteristics.

The path coefficient analysis suggests that more recently released cultivars are less affected by elevated O_3_ concentration in terms of grain number per spike and 1,000-grain weight (**Table 2**). However, the variation in spike number among wheat cultivars released in different years was inconsistent, and the impact on spike number did not reach a significant level. With the replacement of wheat cultivars in the middle and lower reaches of the Yangtze River, the effect on 1,000-grain weight and the number of grains per spike gradually decreased with the elevated O_3_ concentration, whereas the change of spike number was unstable. Path analysis further revealed that the decrease in wheat yield under the elevated O_3_ concentration is mainly attributed to the decrease in grain number per spike and 1,000-grain weight. The decrease in grain number per spike of cultivars released in the 1970s has the greatest effect, but the decrease in 1,000-grain weight of cultivars released since the 1980s is the main factor for yield reduction, likely related to the change of time and elevated O_3_ concentration. Based on these findings, future breeding efforts aiming to improve the tolerance of wheat cultivars to increasing atmospheric O_3_ concentration should primarily focus on stabilizing or increasing the 1000-grain weight (Zhu et al., 2011; Xu et al., 2024).

### 4.3 The effect of O_3_ on the quality of wheat grain

Temperature, light, water, and gas are crucial factors that influence crop growth. Higher temperatures resulting from global warming will reduce wheat yields and quality (Wang et al., 2016). Since the 1950s, the primary wheat cultivars in the middle and lower reaches of the Yangtze River region of China have undergone continuous replacement and iteration, resulting in significant advantages in grain production through genetic improvement (Ding et al., 2020).

Most previous studies on increasing O_3_ concentration have concentrated on the levels of starch, protein, and trace elements, with limited attention given to grain milling quality (Borkowska and Grundas, 2007). The bulk density of grain not only reflects the density and compactness of the grains but is also closely associated with the quality, nutritional value, and processing characteristics of the wheat. Hardness is an intrinsic attribute that determines milling suitability and final application (Erkinbaev et al., 2019). Various cultivation practices and climatic environments can impact wheat milling quality (Borkowska and Grundas, 2007). In this study, we observed that O_3_ significantly reduced the bulk density and hardness of wheat cultivars released over three different decades, although modern cultivars were less affected. Moreover, our unpublished data indicate that modern cultivars show minimal changes in volume, which may contribute to their relatively limited impact on grain weight. Surma et al. (2012) suggest that environmental factors never affect hardness and that only the wheat genotype can influence this trait. However, other research indicates that environmental conditions following flowering can significantly impact physical properties, such as milling yield (Guttieri et al., 2001). Based on the findings of this study, we concluded that the quality characteristic of hardness is influenced by O_3_. Furthermore, wheat seeds subjected to O_3_ fumigation undergo chemical structural changes due to oxidation, which leads to a decrease in the energy required for milling (Desvignes et al., 2008). Additionally, elevated CO_2_ concentration and low nitrogen levels can also contribute to a reduction in grain hardness (Erbs et al., 2010).

Sedimentation value serves as a crucial indicator for evaluating the quality of wheat gluten and protein content, and determining the suitable processing applications for wheat (Liu et al., 2017). In this study, the sedimentation values of various wheat cultivars under O_3_ stress were found to be higher than those under normal atmospheric conditions, which may be related to the increased protein content, given the correlation between sedimentation value and protein levels (Behera et al., 2000). Previous reports indicate that extreme temperature fluctuations during the grain-filling period can lead to a significant decrease in sedimentation value (Labuschagne et al., 2009). Additionally, a study examining the impact of harvest timing on sedimentation value found that delayed harvest times resulted in lower sedimentation values (Ceseviciene and Masauskiene, 2008). Based on these findings, we can infer that elevated O_3_ concentration accelerates the aging of wheat, reducing the length of the growing period and leading to earlier maturation. This could help explain the phenomenon of increased sedimentation values under O_3_ stress. Our results also indicate that the release year of cultivars significantly influenced grain bulk density and sedimentation value, clearly demonstrating that cultivar updates and iterations have a substantial impact on processing quality.

In this study, we observed that elevated O_3_ concentration significantly reduced the starch content in wheat grains, a finding that confirms results from many prior studies (Bhatia et al., 2012; Tomer et al., 2015; Wang YX. et al., 2012). This reduction is primarily due to the considerable impact of O_3_ on photosynthesis, which limits the assimilation and transport of carbon, thus decreasing the amount of sugars and starch transported to the grains (Wang et al., 2012). Additionally, some research indicates that accelerated aging in plants may shorten the time available for carbohydrate formation (Wang and Frei, 2011; Wang et al., 2022), whereas a decrease in the activity of certain starch synthases also affects starch content (Zhang et al., 2013). In our study, the impact of O_3_ on starch content was consistently negative across cultivars released in different decades, with a more pronounced decline observed in those released after 2000. Notably, there was no significant difference in the rate of decline between cultivars from different decades, suggesting that all cultivars are experiencing varying levels of stress.

The impact of elevated O_3_ concentration on grain protein content whether it leads to enhancements or reductions has been extensively debated in previous research (Wang YX. et al., 2012; Tomer et al., 2015; Yadav et al., 2020). The results of this study indicate that cultivars released after 2000 consistently show elevated protein content, whereas those released before that year demonstrate both increases and decreases. Consequently, the effect of elevated O_3_ concentration on grain protein content lacks a clear conclusion, likely due to genotype differences. Yadav et al. (2020) suggested that older cultivars experience a more pronounced decline in protein content, primarily due to changes in the composition of free amino acids and proteins, which aligns with our findings. The increase in protein content observed in modern cultivars may be linked to the acceleration of crop maturation due to O_3_, which reduces grain-filling time and ultimately leads to decreased accumulation of carbohydrates such as starch, thereby raising protein levels (Wang YX. et al., 2012; Li et al., 2021). Furthermore, O_3_ significantly affects nitrogen accumulation in wheat, which also impacts grain protein content. While elevated O_3_ concentration can result in increased grain protein levels, the resulting yield loss far outweighs any nutritional benefits, leading to an overall negative effect on quality. Although O_3_ significantly raises protein content in cultivars released after 2000, this is not necessarily beneficial for specialized wheat cultivars designed for specific uses. This suggests that modern cultivars may be more severely affected by O_3_ stress, a concern that warrants closer attention from researchers.

### 4.4 Implications: recommendations for future O_3_-adapted wheat breeding and O_3_ change prediction models

In the face of climate change, numerous regions and nations have initiated a variety of experiments to address the growing challenge of rising O_3_ concentration (Wang XK. et al., 2012; Yadav D. et al.,2019; Hong et al., 2020; Chaudhary and Rathore, 2022; Naaz et al., 2023). The diverse environmental conditions across these regions underscore the importance of strategic cultivar selection as a crucial aspect of these efforts. Conventionally, trials tend to favor the use of widely grown contemporary cultivars, which are practical and well researched (Pandey et al., 2019; Saitanis et al., 2014). Nevertheless, it is important to acknowledge the possibility that with the march of time and breeding advancements, the current cultivars may become obsolete, overshadowed by future cultivars endowed with superior traits. According to the conclusion that the yield composition is affected by atmospheric O_3_ concentration, O_3_ mainly affects the 1,000-grain weight of wheat. Therefore, it is recommended that breeding research aimed at adapting to climate change should prioritize the development of cultivars with greater grain weight. Efforts should concentrate on breeding wheat with robust grain-filling abilities and employing spike fertilizers to encourage the formation of heavier grains.

Additionally, our findings indicate that wheat yield has increasingly adapted to elevated O_3_ concentration through continuous breeding, showcasing enhanced resistance to O_3_. However, the impact on quality deserves more attention. This insight is pivotal for refining climate change models going forward. Previous models for estimating wheat yield deficits primarily focused on fluctuations in O_3_ levels, overlooking the inherent adaptive potential of the cultivars. By recognizing the inherent adaptability of wheat cultivars and accordingly enhancing flux models, predictions can be rendered with greater precision.

## 5 Conclusion

Our field study in eastern China demonstrated a significant reduction in wheat grain yield due to the elevated O_3_ concentration. As the release year of the cultivars progressed, their tolerance to atmospheric O_3_ was enhanced, leading to a slowdown in yield loss. This suggests that the Yangmai series cultivars can adapt to the prevailing O_3_ concentration at the time of release and develop relative resistance. These cultivars could serve as promising parent choices for future breeding programs focused on developing O_3_-resistant wheat. The results of the path analysis indicate that grain weight has a significant direct impact on yield. Hence, future breeding efforts must prioritize enhancing the 1,000-grain weight to adapt to the detrimental effects of O_3_ stress. Our findings also indicate that elevated O_3_ concentration led to higher sedimentation values and protein content whereas grain weight, hardness, and starch content decreased. The impact on the quality of modern cultivars is particularly pronounced, with significant effects observed on their nutritional properties due to O_3_ stress.

However, this study has certain limitations: it remains unclear whether the regularities observed in the Yangmai series cultivars are applicable to other wheat cultivars, and whether the results hold for wheat grown in different climatic and ecological environments. Additionally, prediction models should incorporate the adaptability of different cultivars to improve accuracy in assessing the impact of O_3_ concentration.

## Data Availability

Data will be made available on request. Requests to access the datasets should be directed to XZ, xkzhu@yzu.edu.cn.
